# Use of the QIAGEN GeneReader NGS system for detection of KRAS mutations, validated by the QIAGEN *Therascreen* PCR kit and alternative NGS platform

**DOI:** 10.1186/s12885-017-3328-z

**Published:** 2017-05-22

**Authors:** Agus Darwanto, Anne-Mette Hein, Sascha Strauss, Yi Kong, Andrew Sheridan, Dan Richards, Eric Lader, Monika Ngowe, Timothy Pelletier, Danielle Adams, Austin Ricker, Nishit Patel, Andreas Kühne, Simon Hughes, Dan Shiffman, Dirk Zimmermann, Kai te Kaat, Thomas Rothmann

**Affiliations:** 1 QIAGEN Waltham, 35 Gatehouse Dr, Waltham, MA 02451 USA; 2 QIAGEN Arhus, Silkeborgvej 2, 8000 Aarhus, Denmark; 3 QIAGEN GmbH, QIAGEN Strasse 1, 40724 Hilden, Nordrhein-Westfalen Germany; 4 QIAGEN Redwood City, 1700 Seaport Blvd, Redwood, CA 94063 USA; 5 QIAGEN Frederick, 6951 Executive Way, Frederick, MD 21703 USA; 6 QIAGEN Manchester, Skelton House Lloyd Street North, Manchester, M15 6SH UK; 7 Novartis Institutes for BioMedical Research, Cambridge, MA 02139 USA; 8 T2 Biosystems, Lexington, MA 02421 USA; 9 Macherey-Nigel, Bethlehem, PA 18020 USA

**Keywords:** GeneReader, Kras, Mutation, Cancer, Ngs

## Abstract

**Background:**

The detection of somatic mutations in primary tumors is critical for the understanding of cancer evolution and targeting therapy. Multiple technologies have been developed to enable the detection of such mutations. Next generation sequencing (NGS) is a new platform that is gradually becoming the technology of choice for genotyping cancer samples, owing to its ability to simultaneously interrogate many genomic loci at massively high efficiency and increasingly lower cost. However, multiple barriers still exist for its broader adoption in clinical research practice, such as fragmented workflow and complex bioinformatics analysis and interpretation.

**Methods:**

We performed validation of the QIAGEN GeneReader NGS System using the QIAact Actionable Insights Tumor Panel, focusing on clinically meaningful mutations by using DNA extracted from formalin-fixed paraffin-embedded (FFPE) colorectal tissue with known KRAS mutations. The performance of the GeneReader was evaluated and compared to data generated from alternative technologies (PCR and pyrosequencing) as well as an alternative NGS platform. The results were further confirmed with Sanger sequencing.

**Results:**

The data generated from the GeneReader achieved 100% concordance with reference technologies. Furthermore, the GeneReader workflow provides a truly integrated workflow, eliminating artifacts resulting from routine sample preparation; and providing up-to-date interpretation of test results.

**Conclusion:**

The GeneReader NGS system offers an effective and efficient method to identify somatic (KRAS) cancer mutations.

**Electronic supplementary material:**

The online version of this article (doi:10.1186/s12885-017-3328-z) contains supplementary material, which is available to authorized users.

## Background

Somatic mutations in the KRAS oncogene are common in human cancers. They are found in 70-90% of pancreatic cancers [1, 2], 30-50% of colorectal cancers [3–5] and 10-30% of Non-Small Cell Lung Cancers (NSCLC) [6–8]. Several methods have been developed for the detection of KRAS mutations, each with specific advantages and limitations [5, 9, 10].

Sanger sequencing has been the ‘gold standard’ for mutation analysis in cancer detection since the 1970s [11]. However, limited by its low sensitivity (10-20% mutant allele frequency (MAF)) and low throughput [10], Sanger sequencing is no longer sufficient for the needs of today’s cancer molecular diagnostics.

The *therascreen* KRAS RGQ PCR kit is a real-time qPCR-based assay used to detect the most common KRAS mutations including those in codons 12 and 13. It has greatly improved sensitivity over Sanger sequencing, and has been approved by the Food and Drug Administration (FDA) [9] for colorectal cancer patient stratification. Pyrosequencing also offers an attractive alternative to Sanger due to its fast turnaround time (TAT) and lower sensitivity threshold, even in tissues with low tumor cell content [5].

Next-generation sequencing (NGS) differs radically from the above mentioned methods. Coupled with amplicon-based targeting technology, NGS has the capability to simultaneously sequence in a massively parallel way multiple genetic loci with minimal amounts of nucleic acid input and limited time and expense [12–15]. This technology has revolutionized the speed of genetic and genomic discovery, and advanced our understanding of molecular mechanisms of diseases. In recent years, NGS has played an important role in advancing personalized healthcare and precision medicine by enabling the identification of mutations associated with therapeutic response or resistance. As more clinically significant genetic biomarkers and targeted therapies become available, the profiling of such genetic variations is becoming increasingly more critical. Several NGS platforms are already commercially available for sequencing and identification of genetic alterations associated with diseases, such as point mutations, deletions, insertions and copy number variants [16]. However, QIAGEN’s GeneReader System presented here includes all upstream sample processing steps starting from nucleic acid extraction, together with an integrated downstream bioinformatics solution that enables a direct access to real-time updates from the rapidly evolving literature, and clinical knowledge and evidence.

To this end, we recently evaluated the QIAGEN GeneReader System workflow from DNA extraction and purification from FFPE tissue samples, to library preparation, sequencing and data analysis and interpretation. Herein we show that the GeneReader presents a unified workflow that provides accurate results and a simple solution for any laboratory to use in clinical research.

## Methods

### Sample and DNA isolation

FFPE Tumor material from colorectal cancer tumors (Origene Technologies, MD, USA and Asterand Biosciences, MI, USA) was used to prepare 56 DNA samples with known KRAS mutation status, previously determined using *therascreen* assay (Pyrosequencing and PCR) and Sanger sequencing according to methods further described below. Tissue sections of 10 μm in thickness, ranging from 3 to 20 years of age were used for DNA extraction utilizing either: i) the QIAamp DNA FFPE Tissue Kit (QIAGEN, Hilden, Germany) or ii) the GeneRead FFPE DNA Kit (QIAGEN, Hilden, Germany) according to manufacturer’s instructions. DNA concentration was determined using the Nanodrop System (Thermo Fisher Scientific, MA, USA) and Qubit dsDNA HS assay (Life Technologies, Gaithersburg, USA). The DNA was assessed using the GeneRead DNA QuantiMIZE System (QIAGEN, Hilden, Germany) which utilizes a qPCR-based approach to determine the quality of sample DNA prior to NGS. Furthermore, both NA12878 (Coriell Institute for Medical Research) (for which the Genome in the Bottle (GIAB) consortium has published a set of high confident variants [17]) and AcroMetrix (Thermo Fisher Scientific, MA, USA) samples were used as a gold standard set of variant calls.

### GeneReader sample preparation and sequencing run

In total, 40 ng of DNA measured by Qubit (Thermo Fisher Scientific, MA, USA) was used as template to generate libraries for sequencing. Libraries were prepared using the QIAGEN Library Kit v2.0 and the GeneRead QIAact Actionable Insight Tumor Panel (QIAGEN, Hilden, Germany), which amplifies 330 amplicons covering 16.7 kb, containing 773 unique variant positions in 12 genes (KRAS, NRAS, KIT, BRAF, PDGFRA, ALK, EGFR, ERBB2, PIK3CA, ERBB3, ESR1 and RAF1). All steps of library preparation were performed according to the manufacturer’s protocol. The libraries were then quantified using a Qubit dsDNA HS Assay Kit (Life Technologies, MA, USA) and QIAxcel (QIAGEN, Hilden, Germany). Ten individual libraries were pooled prior to emulsion PCR and bead enrichment steps that were carried out using an automated protocol on the GeneRead QIAcube (QIAGEN, Hilden, Germany) using the GeneRead Clonal Amp Q Kit (QIAGEN, Hilden, Germany), according to the manufacturer’s protocol. Following bead enrichment, the pooled libraries were sequenced using the GeneReader platform (QIAGEN, Hilden, Germany).

### GeneReader data processing

QIAGEN Clinical Insight **(**QCI™) Analyze software (QIAGEN, Hilden, Germany) was used to QC, align the read data to the hg19 reference genome sequence, call sequence variants, and generate an interactive report for visualization of the sequencing results, as well as a summary of the data. QCI Analyze software reports a set of high- and low-confidence variants based on the coverage of variant positions. Users have an option to analytically confirm if a variant listed should be valid or invalid before uploading to QCI Interpret software for the clinical interpretation. For each sample the report was used to assess the quality of the overall sequencing run and to identify/call the individual variants. After review, variants confirmed as analytically valid were uploaded to QCI Interpret for creation of a report for each sample based on detected variants and curated content, with a summary of findings and direct links to evidence sources.

### Illumina MiSeq

The Actionable Insight Tumor Panel (QIAGEN, Hilden, Germany) was used for a MiSeq (Illumina, CA, USA) sequencing run. The Kapa “with bead” PCR free protocol (KAPABiosystems, MA, USA) was used in further Illumina library preparation steps. Samples were then paired-end sequenced on a MiSeq instrument (Illumina, CA, USA) according to Illumina guidelines. The resulting reads were mapped to the hg19 reference genome sequence using BWA mem software followed by GATK (best practices) to recalibrate base quality scores. Variants were called using MuTect. Variants were then filtered using GATK (best practice) and annotated using SnpEff. Variants at hotspot positions were selected using GATK.

### Pyrosequencing and Sanger analyses

The sample DNA obtained with the QIAamp FFPE DNA Kit (QIAGEN, Hilden, Germany) was subjected to Pyrosequencing analysis and Sanger sequencing. For Pyrosequencing the samples were analyzed using the *therascreen* RAS Extension Pyro Kit (QIAGEN, Hilden, Germany) which covers mutations in KRAS codons 59, 61, 117 and 146 as well as NRAS codons 59, 117 and 146. Samples with mutations in KRAS or NRAS codons 12 and 13 were further analyzed with the *therascreen* KRAS or NRAS Pyro Kit (QIAGEN, Hilden, Germany) according to manufacturer’s instructions. In addition, samples that failed the initial PyroMark KRAS analysis were subjected to a second round of analysis. Samples with an initial “check” status, or with an indicated mutation signal of LOD + 3% (“Potential low level mutation”) were subjected to a second round of analysis performed in duplicate. Sanger sequencing was performed using Big Dye Terminator Technology and an ABI 3730xl sequencer (Thermo Fisher Scientific, MA, USA). Mutations were detected by analyzing the sequence trace files and the quantity of a base at a certain position was calculated from the area under the curve (AUC) at the mutation specific position in the electropherogram.

#### Therascreen qPCR

The *therascreen* KRAS RGQ PCR Kit (QIAGEN, Hilden, UK) is an allele-specific PCR-based technology with specific primers for the seven most common KRAS codon 12 and 13 mutations. The assay screens for the following mutations: 12 GCT (Ala), 12 GAT (Asp), 12 CGT (Arg), 12 TGT (Cys), 12 AGT (Ser), 12 GTT (Val), and 13 GAC (Asp). Mutation analysis was performed according to manufacturer’s instructions, using the RotorGene real-time PCR instrument (QIAGEN, Hilden, UK). Analysis of results was performed following the recommendations in the manual, e.g. samples with a control assay with a cycle threshold (Ct) of 35 or higher were deemed invalid and excluded from the analysis. Samples were called mutation positive based on the delta Ct values reported in the handbook. Values over 40 cycles were scored as negative (wild-type).

## Results

### Evaluation of DNA quality by QuantiMIZE

FFPE samples with ages ranging from 3 to 20 years were used for this study. The quality of the extracted DNA was measured by the GeneRead DNA QuantiMIZE QC assay (QIAGEN, Hilden, UK). Thirteen out of 56 samples failed quality checks and were excluded from further analysis (Additional file 1: Table S1). For the remaining 43 samples, 3 to 9 PCR cycles were added (depending on the QuantiMIZE quality scores) to compensate for differences in DNA quality during enrichment PCR. The additional cycles ensured that poor quality (highly fragmented) DNA samples yielded enough material for downstream library preparation. The quality of DNA purified from formalin fixated tissue decreases over the sample storage period time [18–20], but also depends on how tissues were treated, handled and processed before and during sample fixation [19, 21, 22].

### GeneReader sequencing performance

The QIAact Actionable Insights Tumor Panel (QIAGEN, Hilden, UK) contains 773 unique variant positions in 12 genes (Table 1). An analysis of the reads mapped to the reference showed coverage levels that met the industry-standard 5% sensitivity criteria, even with aged FFPE samples. A 200× minimum read coverage cutoff was used for calling a variant at any position in the panel. For the 43 FFPE samples analyzed, an average amplicon coverage of 97.2% was observed, and an average variant insight coverage (hotspot coverage) of 99.8% was observed at read depths ≥200× (Table 1). For NA12878 samples, an average amplicon coverage of 98.5% was observed and an average variant insight coverage of 99.9% was observed at read depths of ≥200× (Table 1). No false negatives (FN; where an expected variant was not detected) were observed.

**Table 1:** Parameter and sequencing coverage of Actionable Insight Tumor Panel

Parameter	Details
Panel size	12 genes/16.7 kb
Insight size	773 unique variant positions
Amplicons	330
Variant allele fraction detection limit	5%
Frequency cut-off and amplicon coverage	>500×: 96.4% (A), 92.0% (B)
	>200×: 98.5% (A), 97.2% (B)
Frequency cut-off and variant insight coverage	>500×: 99.8% (A), 98.6% (B)
	>200×: 99.9% (A), 99.8% (B)

Positive samples included into the study have all been confirmed with Sanger sequencing and passed QuantiMIZE (<0.4). (A) An average of 12 NA12878 samples, (B) average of 43 colorectal cancers FFPE samples (ages 3-20 years)

### Performance comparison between the QIAamp and GeneRead DNA FFPE kits for DNA purification using the GeneReader

Two DNA purification kits were used to isolate DNA from FFPE samples. Table 2 demonstrates the superior performance of the GeneRead DNA FFPE Kit (QIAGEN, Hilden, UK) over the QIAamp DNA FFPE Tissue Kit (QIAGEN, Hilden, UK) in terms of true positives at lower variant calling sensitivity. Fourteen true positive KRAS variants were detected using an allele fraction cut-off of >5% for DNA isolated by GeneRead DNA FFPE Kit (QIAGEN, Hilden, UK). For the QIAamp DNA FFPE Tissue Kit (QIAGEN, Hilden, UK), 15 KRAS variants were detected using an allele fraction cut-off of >5%. Of the 15 KRAS variants detected, 14 were true positive variants and 1 was a false positive (Table 2) as confirmed by several independent methods. Decreasing the allele fraction cutoff to >2.5% resulted in identification of the same 14 KRAS true positive samples for GeneRead DNA FFPE Kit (QIAGEN, Hilden, UK) extractions. However, for QIAamp DNA FFPE Tissue Kit (QIAGEN, Hilden, UK) extracted samples at >2.5% allele fraction cut-off, 11 additional false positive KRAS mutations (25 variants in total) were detected. The additional mutations were mostly C to T transitions. It is known that FFPE fixation deaminates certain bases, most prominently cytosine deamination to uracil [23–25]. The GeneRead DNA FFPE Kit (QIAGEN, Hilden, UK) contains an integrated uracil DNA glycosylase (UDG) step which removes uracil from the DNA before the final purification step, yielding high-quality DNA with minimal artifacts.

**Table 2:** The GeneReader FFPE DNA sample preparation kit successfully corrects FFPE artifacts

Type of DNA purification kit	Allele frequency cut off	
	>5%	>2.5%
QIAamp FFPE DNA purification Kit	15	25
GeneRead DNA FFPE Kit	14	14

### Confirmation of variants by MiSeq, pyrosequencing and *therascreen* qPCR assays

The GeneReader NGS System variant calls demonstrated 100% agreement with KRAS mutation status previously determined by either pyrosequencing or *therascreen* qPCR (Table 3). Of the 43 samples, 14 tested positive for KRAS variants and 29 samples were confirmed as wild type. The 5% allelic fraction cut-off was used to call KRAS variants for codons 12, 13, 59, 61, 117 and 146. The true positive variants observed by the GeneReader NGS System share a 100% concordance with MiSeq-Illumina (Table 4).

**Table 3:** KRAS agreement study between GeneReader and Pyrosequencing and *Therascreen* PCR Assays

>5% KRAS variant allele frequency cut off		Pyrosequencing and *Therascreen* PCR Assays^(a)^		
		(MT)	(WT)	Total
GeneReader NGS System^(b)^	(MT)	14	0	14
	(WT)	0	29	29
	Total	14	29	43

^(a)^If KRAS is mutant by *Therascreen* KRAS RGQ PCR assay or *Therascreen* RAS extension Pyrosequencing assay, the condition is recorded as a mutant (MT)

^(b)^For Actionable Insights Tumor Panel, a 5% allelic frequency cut off was used to call variants for codon 12, 13, 59, 61, 117 and 146, which are addressed by established QIAGEN*Therascreen* PCR assays

**Table 4:** The concordance study between GeneReader, MiSeq, Pyrosequencingand *Therascreen* PCR assays

Sample no.	KRAS AA change	KRAS variant allele fraction (%)		
		*Therascreen* PCR/Pyro	GeneReader^a^	MiSeq^a^
1	G12D	+	21	7
2	G12D	+	39	12
3	A59T	19	15	14
4	G13D	+	41	15
5	G12D	+	45	11
6	Q61H	14	9	13
7	A146P	41	40	32
8	Q61H	36	35	26
9	Q61H	32	22	35
10	K117 N	24	34	39
11	G13D	+	47	15
12	G12C	+	32	10
13	G13D	+	39	10
14	Q61H	26	21	23

+: Variant identified by *Therascreen* PCR; allele fraction not available

^a^: Sample processed from different FFPE section with potentially different tumor content and variant allele fraction

**Fig. 1:**
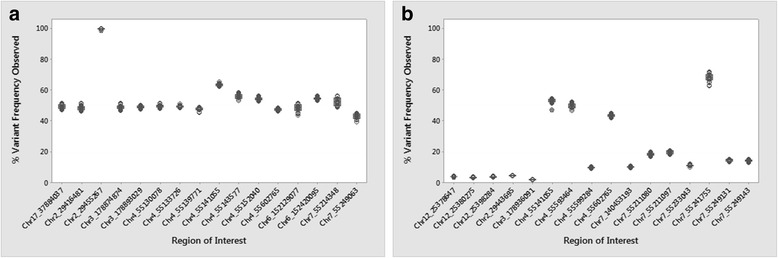
Variant calling performances of GeneReader pipeline. Each individual data point was generated from 18 data points (**a**) NA12878 and (**b**) AcroMetrix Oncology Hotspot

The use of the NA12878 control (Fig. 1, Additional file 2: Table S2) and AcroMetrix (Fig. 1, Additional file 3: Tables S3) reference standard materials demonstrated the good performance of the GeneReader platform on high frequency and low frequency variants, respectively. NA12878 has been used extensively as a reference standard material for verifying NGS platforms [17] and acts as a useful control in establishing background error. Besides its use as a GeneReader platform performance standard, AcroMetrix has also been used previously as a control for variant calls [26].

## Discussion

A major advantage of NGS over traditional mutation detection methods is the ability to sequence multiple genes and variants simultaneously. Other advantages include minimal DNA input, faster turnaround time; lower overall cost and higher throughput and sensitivity compared to traditional methods [12, 27–29]. NGS has revolutionized the speed of genetic and genomic discovery, and advanced our understanding of the molecular mechanisms of disease and potential treatment options. However, several major hurdles remain and still prevent NGS from being broadly adopted in clinical practice. This is especially true for laboratories that are new to this technology, and may lack the in-house expertise required for processing complex bioinformatics data and interpretation of results. Such expertise is crucial to construct a bioinformatics pipeline and to evaluate the software and generate quality reports. The QIAGEN GeneReader NGS System allows users to perform experiments from sample to insight, tissue sample to decipherable report based on the interpretation of sequence variants detected.

**Fig. 2:**
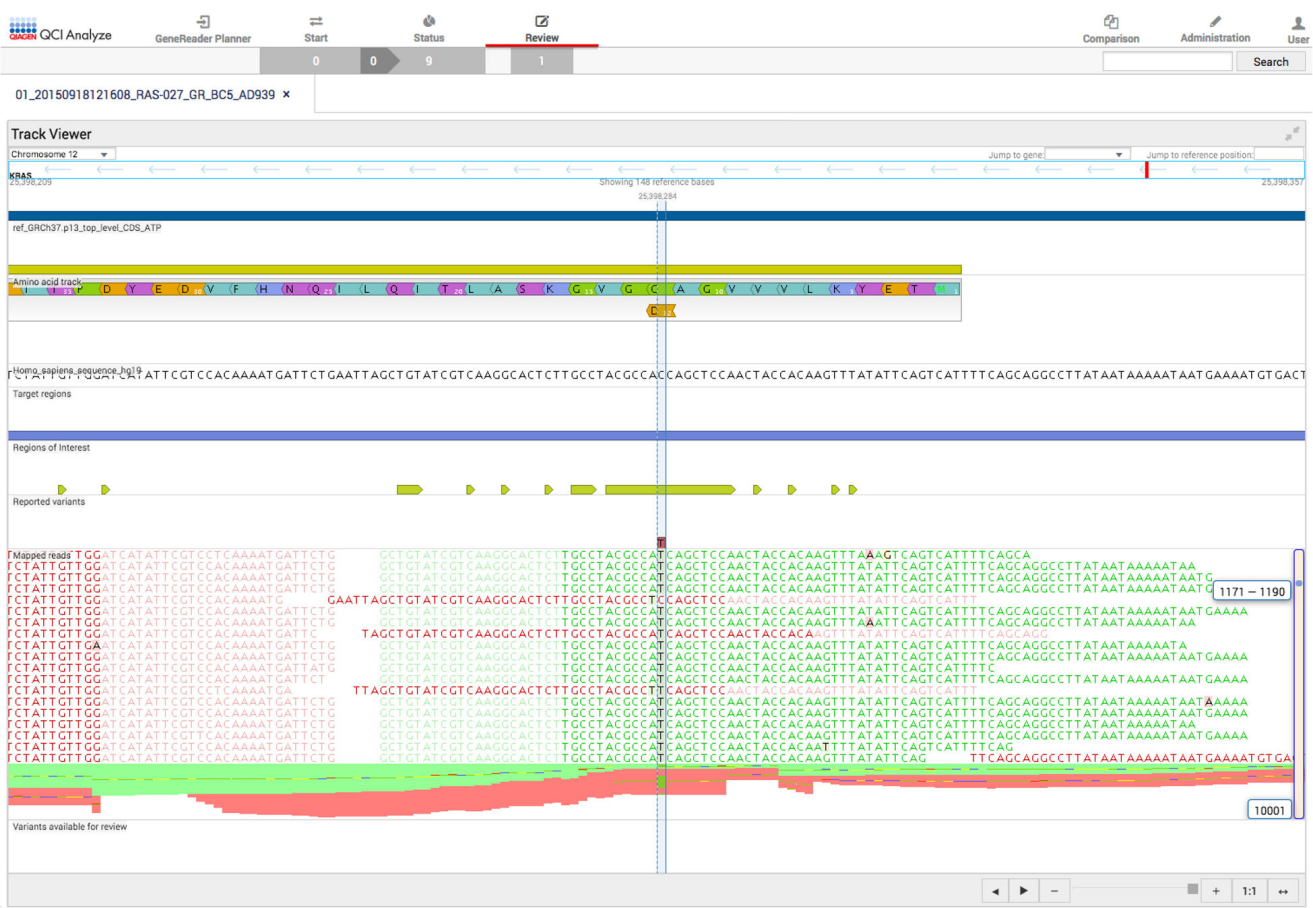
QCI Analyze report showing the alignment of the reads at the variant positions along with the induced amino acid change

**Fig. 3:**
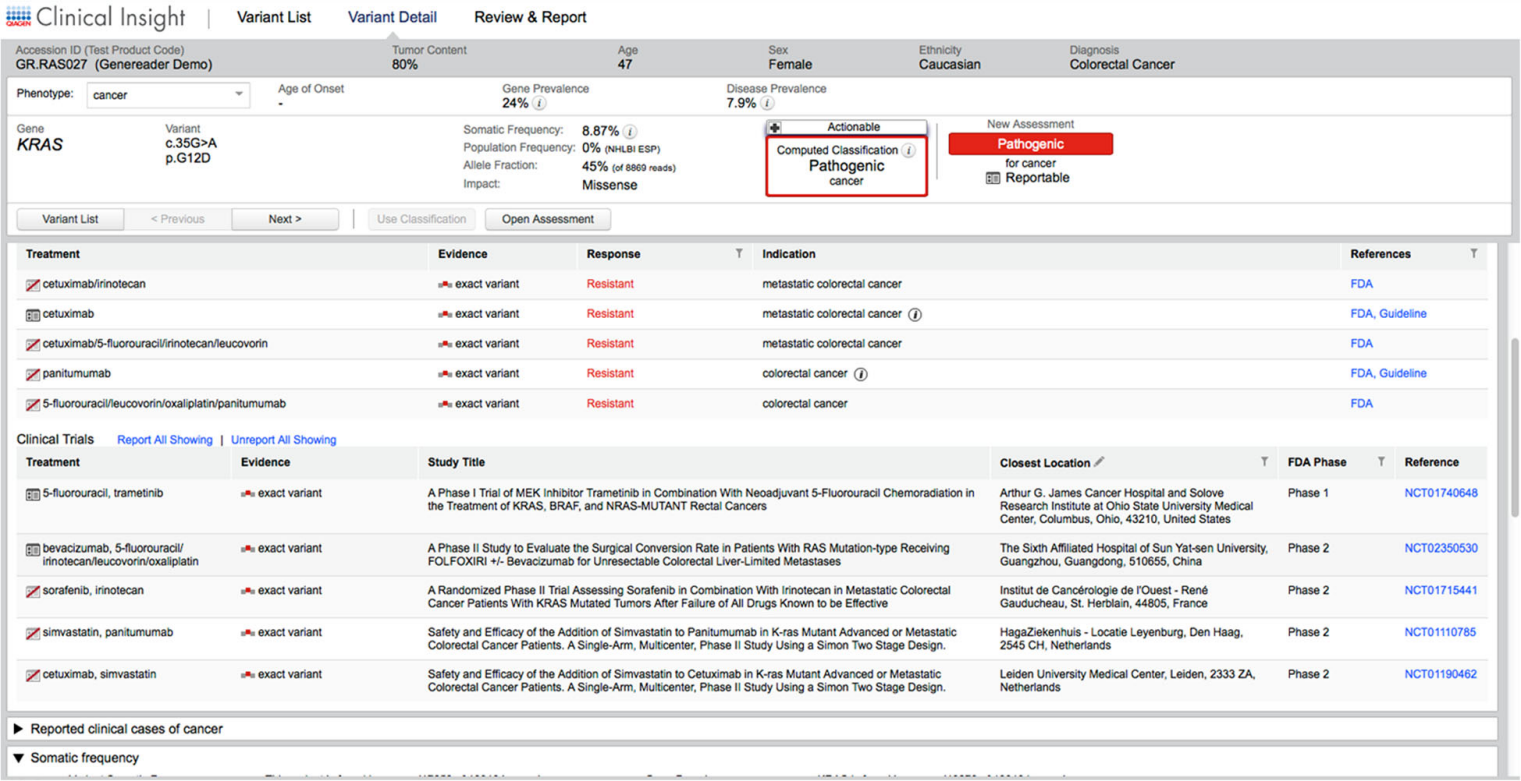
QCI Interpret actionable report, showing summary of findings and link to the insights that can be used to guide clinical research

The QIAGEN GeneReader NGS workflow utilizes ‘QCI Analyze’ and ‘QCI Interpret’ for bioinformatics analysis and reporting of variants, including read mapping, variant calling and interpretation of results. It provides visualization of the alignment of sequencing results (Fig. 2) as well as a summary of the data. Quality assessment is also supported, both at the overall sequencing run level and for the analytic validity of individual variants to reduce false positive and negative results. Using the data visualization tools within QCI Analyze, it is possible to determine the quality of the results and assess any variants of interest. Further analysis of variants using QCI Interpret provides access to the curated information contained within the QIAGEN Knowledge Base enabling a deeper analysis and interpretation of results for each sample (Fig. 3). With all relevant information, a report can be created with a summary of findings and direct links to evidence sources. At the single variant level the QCI software is able to identify an individual variant as an actionable cancer mutation, and provides links to current clinical research insights, e.g. the KRAS G12D somatic variant it is established to confer resistance to the colorectal cancer drugs cetuximab and panitumumab, based on evidence curated from their FDA drug labels and clinical practice guidelines. Within QCI-Interpret information on active clinical trials recruiting colorectal cancer patients with particular mutations are provided with drug, nearest location, and trial phase information.

The relationship between FFPE DNA quality and sequencing accuracy is a critical point for any sequencing analysis. The GeneReader workflow starts with the GeneRead FFPE DNA Kit for DNA extraction and is specifically designed to reduce artifacts known to commonly occur in FFPE treated samples. As seen in Table 2, by using FFPE samples aged from 3 to 20 years, the GeneRead FFPE DNA Kit successfully reduced the number of low frequency false positive variants detected. These low frequency false positive variants are likely caused by cytosine deamination and other fixation associated artifacts. Similar phenomena were observed by Bourgon [23], where pretreatment of FFPE samples with uracil DNA glycosylase (UDG) resulted in a dramatic reduction of false positives, with overall reductions of 77% for C > T and 94% for G > A changes, respectively. Biochemical removal of deaminated DNA eliminates deamination-associated false positive results; however, for samples with very low quality DNA such as highly fragmented FFPE treated samples, UDG-treated may constitute an issue, as the treatment introduces possible further strand breaks leading to even higher fragmentation and lower availability of intact template strands. Therefore, using the QuantiMIZE assay to identify those samples suitable for sequencing, based on an assessment of original intact and amplifiable templates, before starting an experiment is a critical point for an amplification based NGS technology. Previous reports observed that samples with lower amounts of amplifiable DNA are more likely to give a markedly increased number of false positive results [30, 31].

## Conclusions

In summary, this study confirms that the GeneReader NGS System performs consistently and accurately in the identification of somatic mutations from FFPE samples, with results confirmed by both alternative technologies as well as an alternative NGS platform. With a full end-to-end solution with integrated sample preparation and bioinformatics interpretation, the GeneReader NGS System is suitable for any laboratory interested in cancer clinical research.

## Additional files

Additional file 1: Table S1. The QC results of the extracted DNA samples were measured using GeneRead DNA QuantiMIZE. (DOCX 28 kb)

Additional file 2: Table S2. List of NA12878 Gold Standard Variants from 18 samples sequenced by GeneReader. (DOCX 27 kb)

Additional file 3: Table S3. List of AcroMetrix™ Oncology Hotspot Gold Standard Variants from 18 samples sequenced by GeneReader (DOCX 28 kb) (DOCX 27 kb)

## Abbreviations

Ct: Cycle threshold
DNA: Deoxyribonucleic acid
dsDNA: Double-stranded DNA
FDA: Food and Drug Administration
FFPE: Formalin-fixed paraffin-embedded
GR: GeneReader
NGS: Next generation sequencing
NSCLC: Non-small cell lung cancer
PCR: Polymerase chain reaction
qPCR: Quantitative PCR
QC: Quality control
QCI: QIAGEN clinical insight
TAT: Turn-around time
UDG: Uracil-DNA glycosylase
