# Supplementary material for: Use of the QIAGEN GeneReader NGS system for detection of KRAS mutations, validated by the QIAGEN Therascreen PCR kit and alternative NGS platform
Source: BMC Cancer. 2017 May 22;17:358. doi: 10.1186/s12885-017-3328-z (PMC5441096; doi:10.1186/s12885-017-3328-z)
Supplement: Supplementary file 1 — The QC results of the extracted DNA samples were measured using GeneRead DNA QuantiMIZE. (DOCX 28 kb) [file 12885_2017_3328_MOESM1_ESM.docx]

**Supplementary Table 1:** The QC results of the extracted DNA samples were measured using GeneRead DNA QuantiMIZE.

| Sample ID | QC score | QC Call | Age of sample (years) | Added Cycles | No. of Cycles | Total Sample Volume (µl) | ddH2O (µl) |
| --- | --- | --- | --- | --- | --- | --- | --- |
| RAS-001_GR | 0.05 | Low | 14 | 9 | 30 | 8 | 11 |
| RAS-001_Q | 0.04 | Low | 14 | 9 | 30 | 9 | 10 |
| RAS-002_GR | 0.04 | High | 21 | 7 | 28 | 15 | 4 |
| RAS-002_Q | 0.032 | High | 21 | 7 | 28 | 11 | 8 |
| RAS-003_GR | 0.033 | High | 16 | 5 | 26 | 18 | 1 |
| RAS-003_Q | 0.026 | High | 16 | 5 | 26 | 8 | 11 |
| RAS-006_GR | 0.035 | High | 11 | 7 | 28 | 6 | 13 |
| RAS-006_Q | 0.033 | High | 11 | 5 | 26 | 19 | 0 |
| RAS-007_GR | 0.021 | High | 10 | 3 | 24 | 18 | 1 |
| RAS-007_Q | 0.023 | High | 10 | 3 | 24 | 16 | 3 |
| RAS-010_GR | 0.051 | Low | 10 | 9 | 30 | 12 | 7 |
| RAS-010_Q | 0.032 | High | 10 | 9 | 30 | 19 | 0 |
| RAS-011_GR | 0.041 | Low | 9 | 9 | 30 | 7 | 12 |
| RAS-011_Q | 0.036 | High | 9 | 9 | 30 | 12 | 8 |
| RAS-012_GR | 0.022 | High | 9 | 5 | 26 | 5 | 14 |
| RAS-012_Q | 0.022 | High | 9 | 3 | 24 | 18 | 1 |
| RAS-013_GR | 0.016 | High | 9 | 5 | 26 | 6 | 13 |
| RAS-013_Q | 0.019 | High | 9 | 5 | 26 | 5 | 14 |
| RAS-014_GR | 0.023 | High | 9 | 5 | 26 | 8 | 11 |
| RAS-014_Q | 0.021 | High | 9 | 5 | 26 | 7 | 12 |
| RAS-017_GR | 0.014 | High | 9 | 3 | 24 | 7 | 12 |
| RAS-017_Q | 0.009 | High | 9 | 3 | 24 | 6 | 13 |
| RAS-018_GR | 0.017 | High | 8 | 5 | 26 | 9 | 10 |
| RAS-018_Q | 0.021 | High | 8 | 5 | 26 | 8 | 11 |
| RAS-019_GR | 0.022 | High | 8 | 5 | 26 | 8 | 11 |
| RAS-019_Q | 0.023 | High | 8 | 5 | 26 | 7 | 13 |
| RAS-020_GR | 0.029 | High | 8 | 7 | 28 | 5 | 14 |
| RAS-020_Q | 0.028 | High | 8 | 5 | 26 | 15 | 4 |
| RAS-021_GR | 0.031 | High | 8 | 5 | 26 | 17 | 2 |
| RAS-021_Q | 0.033 | High | 8 | 5 | 26 | 11 | 8 |
| RAS-023_GR | 0.018 | High | 8 | 3 | 24 | 16 | 3 |
| RAS-023_Q | 0.023 | High | 8 | 3 | 24 | 15 | 4 |
| RAS-025_GR | 0.038 | High | 8 | 9 | 30 | 10 | 10 |
| RAS-025_Q | 0.037 | High | 8 | 9 | 30 | 8 | 11 |
| RAS-026_GR | 0.033 | High | 8 | 7 | 28 | 13 | 6 |
| RAS-026_Q | 0.043 | Low | 8 | 9 | 30 | 5 | 14 |
| RAS-027_GR | 0.011 | High | 8 | 3 | 24 | 10 | 10 |
| RAS-027_Q | 0.018 | High | 8 | 3 | 24 | 9 | 11 |
| RAS-028_GR | 0.036 | High | 8 | 9 | 30 | 6 | 13 |
| RAS-028_Q | 0.049 | Low | 8 | 9 | 30 | 11 | 8 |
| RAS-029_GR | 0.014 | High | 8 | 5 | 26 | 6 | 14 |
| RAS-029_Q | 0.025 | High | 8 | 5 | 26 | 5 | 14 |
| RAS-031_GR | 0.024 | High | 8 | 5 | 26 | 10 | 9 |
| RAS-031_Q | 0.024 | High | 8 | 5 | 26 | 8 | 12 |
| RAS-032_GR | 0.023 | High | 8 | 5 | 26 | 5 | 14 |
| RAS-032_Q | 0.018 | High | 8 | 3 | 24 | 13 | 6 |
| RAS-033_GR | 0.033 | High | 7 | 7 | 28 | 12 | 7 |
| RAS-033_Q | 0.03 | High | 7 | 7 | 28 | 8 | 11 |
| RAS-034_GR | 0.028 | High | 6 | 5 | 26 | 16 | 3 |
| RAS-034_Q | 0.033 | High | 6 | 5 | 26 | 14 | 5 |
| RAS-043_GR | 0.018 | High | >3 | 3 | 24 | 18 | 1 |
| RAS-043_Q | 0.024 | High | >3 | 5 | 26 | 6 | 13 |
| RAS-044_GR | 0.035 | High | >3 | 7 | 28 | 6 | 13 |
| RAS-044_Q | 0.029 | High | >3 | 7 | 28 | 6 | 14 |
| RAS-047_GR | 0.024 | High | >3 | 5 | 26 | 10 | 9 |
| RAS-047_Q | 0.031 | High | >3 | 7 | 28 | 10 | 9 |
| RAS-055_GR | 0.038 | High | >3 | 7 | 28 | 10 | 9 |
| RAS-055_Q | 0.033 | High | >3 | 7 | 28 | 14 | 5 |
| RAS-057_GR | 0.03 | High | >3 | 7 | 28 | 5 | 14 |
| RAS-057_Q | 0.033 | High | >3 | 7 | 28 | 9 | 11 |
| RAS-061_GR | 0.031 | High | >3 | 7 | 28 | 6 | 13 |
| RAS-061_Q | 0.04 | High | >3 | 9 | 30 | 6 | 13 |
| RAS-062_GR | 0.03 | High | >3 | 7 | 28 | 7 | 12 |
| RAS-062_Q | 0.052 | Low | >3 | 9 | 30 | 18 | 2 |
| RAS-067_GR | 0.022 | High | >3 | 3 | 24 | 19 | 0 |
| RAS-067_Q | 0.026 | High | >3 | 5 | 26 | 11 | 8 |
| RAS-068_GR | 0.028 | High | >3 | 5 | 26 | 14 | 5 |
| RAS-068_Q | 0.026 | High | >3 | 9 | 30 | 5 | 14 |
| RAS-075_GR | 0.036 | High | >3 | 7 | 28 | 5 | 14 |
| RAS-075_Q | 0.034 | High | >3 | 7 | 28 | 14 | 5 |
| RAS-076_GR | 0.028 | High | >3 | 5 | 26 | 18 | 1 |
| RAS-076_Q | 0.037 | High | >3 | 7 | 28 | 18 | 1 |
| RAS-078_GR | 0.037 | High | >3 | 7 | 28 | 12 | 7 |
| RAS-078_Q | 0.028 | High | >3 | 9 | 30 | 12 | 7 |
| RAS-079_GR | 0.038 | High | >3 | 9 | 30 | 11 | 8 |
| RAS-079_Q | 0.034 | High | >3 | 7 | 28 | 6 | 13 |
| RAS-080_GR | 0.037 | High | >3 | 7 | 28 | 11 | 8 |
| RAS-080_Q | 0.033 | High | >3 | 9 | 30 | 12 | 8 |
| RAS-082_GR | 0.023 | High | >3 | 3 | 24 | 19 | 0 |
| RAS-082_Q | 0.016 | High | >3 | 5 | 26 | 6 | 13 |
| RAS-087_GR | 0.029 | High | >3 | 5 | 26 | 19 | 0 |
| RAS-087_Q | 0.029 | High | >3 | 7 | 28 | 11 | 8 |
| RAS-103_GR | 0.035 | High | >3 | 7 | 28 | 12 | 7 |
| RAS-103_Q | 0.039 | High | >3 | 9 | 30 | 15 | 4 |
| RAS-104_GR | 0.035 | High | >3 | 7 | 28 | 12 | 7 |
| RAS-104_Q | 0.04 | Low | >3 | 9 | 30 | 11 | 8 |
| RAS-106_GR | 0.03 | High | >3 | 7 | 28 | 6 | 13 |
| RAS-106_Q | 0.041 | Low | >3 | 9 | 30 | 9 | 11 |
| RAS-107_GR | 0.035 | High | >3 | 7 | 28 | 17 | 3 |
| RAS-107_Q | 0.035 | High | >3 | 9 | 30 | 13 | 6 |
| RAS-108_GR | 0.037 | High | >3 | 7 | 28 | 9 | 10 |
| RAS-108_Q | 0.028 | High | >3 | 7 | 28 | 9 | 11 |
| RAS-110_GR | 0.062 | Low | 22 | 9 | 30 | 14 | 5 |
| RAS-110_Q | 0.056 | Low | 22 | 9 | 30 | 17 | 2 |
| RAS-113_GR | 0.032 | High | >3 | 5 | 26 | 11 | 8 |
| RAS-113_Q | 0.028 | High | >3 | 7 | 28 | 5 | 14 |
| RAS-114_GR | 0.058 | Low | >3 | 9 | 30 | 9 | 10 |
| RAS-114_Q | 0.048 | Low | >3 | 9 | 30 | 14 | 5 |
| RAS-116_GR | 0.057 | Low | >3 | N/A | N/A | N/A | N/A |
| RAS-116_Q | 0.05 | Low | >3 | N/A | N/A | N/A | N/A |
| RAS-117_GR | 0.036 | High | >3 | 7 | 28 | 6 | 13 |
| RAS-117_Q | 0.027 | High | >3 | 7 | 28 | 6 | 14 |
| RAS-118_GR | 0.036 | High | >3 | 7 | 28 | 5 | 14 |
| RAS-118_Q | 0.029 | High | >3 | 5 | 26 | 17 | 2 |
| RAS-119_GR | 0.044 | Low | >3 | 7 | 28 | 11 | 8 |
| RAS-119_Q | 0.037 | High | >3 | 7 | 28 | 12 | 7 |
| RAS-120_GR | 0.05 | Low | >3 | 9 | 30 | 5 | 14 |
| RAS-120_Q | 0.042 | Low | >3 | 9 | 30 | 7 | 13 |
| RAS-121_GR | 0.02 | High | >3 | 3 | 24 | 16 | 3 |
| RAS-121_Q | 0.015 | High | >3 | 5 | 26 | 6 | 13 |
| RAS-122_GR | 0.04 | Low | >3 | 7 | 28 | 6 | 13 |
| RAS-122_Q | 0.023 | High | >3 | 7 | 28 | 7 | 12 |

**Note**: >3 no exact information of FFPE age, however we purchased from the vendor in 2012 and DNA extractions were performed in 2015.
