# Supplementary material for: Use of the QIAGEN GeneReader NGS system for detection of KRAS mutations, validated by the QIAGEN Therascreen PCR kit and alternative NGS platform
Source: BMC Cancer. 2017 May 22;17:358. doi: 10.1186/s12885-017-3328-z (PMC5441096; doi:10.1186/s12885-017-3328-z)
Supplement: Supplementary file 2 — List of NA12878 Gold Standard Variants from 18 samples sequenced by GeneReader. (DOCX 27 kb) [file 12885_2017_3328_MOESM2_ESM.docx]

**Supplementary Table 2.** List of NA12878 Gold Standard Variants from 18 samples sequenced by GeneReader.

| Chromosome Region | % Variant Frequency Observed | | | | | | | | | | | | | | | | | | Average | SD |
| --- | --- | --- | --- | --- | --- | --- | --- | --- | --- | --- | --- | --- | --- | --- | --- | --- | --- | --- | --- | --- |
|  | S1 | S2 | S3 | S4 | S5 | S6 | S7 | S8 | S9 | S10 | S11 | S12 | S13 | S14 | S15 | S16 | S17 | S18 |  |  |
| Chr2_29416481 | 47.42 | 49.71 | 47.93 | 51.34 | 50.12 | 48.65 | 46.87 | 47.71 | 48.54 | 46.46 | 48.52 | 49.08 | 46.28 | 46.81 | 47.05 | 48.76 | 49.14 | 51.08 | 48.42 | 1.50 |
| Chr2_29455267 | 99.80 | 99.78 | 99.62 | 99.96 | 99.85 | 99.68 | 99.61 | 99.74 | 98.69 | 99.76 | 99.62 | 99.79 | 99.62 | 99.78 | 99.71 | 99.58 | 99.69 | 99.67 | 99.66 | 0.26 |
| Chr3_178874874 | 48.67 | 48.11 | 48.91 | 47.10 | 49.22 | 48.74 | 46.86 | 49.79 | 47.71 | 49.75 | 48.03 | 47.94 | 51.50 | 50.98 | 50.56 | 49.82 | 48.83 | 48.47 | 48.94 | 1.28 |
| Chr3_178893029 | 48.27 | 48.53 | 49.03 | 48.21 | 48.35 | 49.01 | 49.72 | 50.15 | 48.74 | 48.15 | 49.77 | 48.79 | 49.89 | 48.44 | 49.64 | 49.27 | 47.46 | 48.15 | 48.87 | 0.74 |
| Chr4_55130078 | 49.15 | 50.99 | 49.69 | 50.18 | 49.62 | 48.25 | 49.09 | 51.55 | 50.84 | 48.80 | 49.77 | 49.80 | 48.23 | 49.88 | 48.89 | 48.88 | 50.90 | 49.83 | 49.69 | 0.94 |
| Chr4_55133726 | 48.28 | 49.97 | 48.43 | 49.54 | 48.92 | 49.54 | 50.26 | 48.81 | 49.80 | 49.30 | 49.86 | 49.70 | 49.29 | 49.92 | 49.92 | 50.90 | 48.72 | 49.87 | 49.50 | 0.67 |
| Chr4_55139771 | 45.39 | 47.26 | 46.96 | 46.97 | 47.52 | 48.17 | 47.04 | 49.08 | 47.87 | 48.27 | 48.65 | 48.37 | 47.78 | 47.93 | 47.33 | 48.57 | 45.92 | 47.32 | 47.58 | 0.94 |
| Chr4_55141055 | 64.12 | 63.81 | 63.52 | 64.24 | 65.51 | 64.11 | 62.44 | 62.45 | 62.77 | 64.26 | 62.41 | 63.98 | 64.61 | 63.63 | 62.33 | 63.27 | 63.85 | 62.86 | 63.56 | 0.88 |
| Chr4_55143577 | 58.51 | 58.12 | 55.05 | 55.59 | 53.91 | 55.75 | 55.62 | 57.22 | 56.40 | 53.31 | 56.57 | 54.05 | 55.45 | 54.11 | 54.85 | 56.57 | 58.36 | 57.58 | 55.95 | 1.59 |
| Chr4_55152040 | 54.38 | 53.65 | 55.24 | 53.76 | 55.72 | 54.75 | 53.24 | 53.79 | 52.95 | 56.13 | 54.04 | 54.34 | 55.32 | 53.87 | 53.77 | 54.43 | 54.21 | 53.81 | 54.30 | 0.85 |
| Chr4_55602765 | 46.87 | 47.45 | 46.56 | 47.72 | 48.39 | 47.35 | 47.01 | 47.58 | 46.52 | 47.71 | 48.51 | 46.63 | 47.72 | 47.99 | 48.20 | 47.42 | 47.79 | 46.21 | 47.42 | 0.67 |
| Chr6_152129077 | 51.09 | 49.09 | 49.36 | 50.55 | 47.06 | 47.21 | 49.93 | 50.41 | 48.20 | 45.29 | 49.27 | 48.11 | 43.86 | 44.59 | 44.98 | 50.48 | 48.34 | 51.57 | 48.30 | 2.36 |
| Chr6_152420095 | 56.30 | 55.65 | 54.65 | 54.65 | 53.91 | 53.76 | 55.36 | 54.78 | 55.05 | 53.25 | 54.87 | 54.31 | 54.58 | 52.77 | 53.77 | 54.76 | 56.04 | 54.54 | 54.61 | 0.91 |
| Chr7_55214348 | 51.26 | 52.36 | 49.64 | 49.07 | 54.49 | 54.28 | 51.81 | 49.63 | 52.45 | 56.30 | 52.99 | 53.69 | 56.30 | 55.97 | 56.38 | 49.45 | 51.92 | 50.02 | 52.67 | 2.55 |
| Chr7_55249063 | 40.62 | 41.84 | 43.17 | 42.87 | 44.54 | 43.67 | 40.81 | 39.53 | 43.26 | 44.73 | 43.91 | 41.04 | 45.12 | 45.07 | 45.04 | 42.27 | 43.97 | 41.94 | 42.97 | 1.72 |
| Chr17_37884037 | 47.13 | 47.84 | 48.79 | 49.05 | 51.10 | 50.48 | 47.56 | 48.15 | 47.98 | 49.40 | 50.68 | 50.28 | 50.11 | 51.25 | 51.56 | 50.52 | 48.30 | 48.83 | 49.39 | 1.39 |
