# Supplementary material for: Use of the QIAGEN GeneReader NGS system for detection of KRAS mutations, validated by the QIAGEN Therascreen PCR kit and alternative NGS platform
Source: BMC Cancer. 2017 May 22;17:358. doi: 10.1186/s12885-017-3328-z (PMC5441096; doi:10.1186/s12885-017-3328-z)
Supplement: Supplementary file 3 — List of AcroMetrix™ Oncology Hotspot Gold Standard Variants from 18 samples sequenced by GeneReader (DOCX 28 kb) (DOCX 27 kb) [file 12885_2017_3328_MOESM3_ESM.docx]

**Supplementary Table 3.** List of AcroMetrix Oncology Hotspot Gold Standard Variants from 18 samples sequenced by GeneReader

| Chromosome Region | % Variant Frequency Observed | | | | | | | | | | | | | | | | | | Average | SD |
| --- | --- | --- | --- | --- | --- | --- | --- | --- | --- | --- | --- | --- | --- | --- | --- | --- | --- | --- | --- | --- |
|  | S1 | S2 | S3 | S4 | S5 | S6 | S7 | S8 | S9 | S10 | S11 | S12 | S13 | S14 | S15 | S16 | S17 | S18 |  |  |
| Chr2_29443695 | 4.73 | 4.60 | 4.23 | 4.36 | 4.73 | 4.83 | 4.52 | 4.56 | 4.44 | 4.41 | 4.76 | 4.51 | 4.51 | 4.40 | 4.16 | 4.79 | 4.63 | 4.45 | 4.53 | 0.19 |
| Chr3_178936091 | 1.88 | 1.97 | 1.99 | 1.93 | 1.64 | 2.02 | 2.02 | 1.90 | 1.95 | 1.86 | 1.82 | 1.80 | 1.98 | 1.88 | 1.99 | 1.82 | 1.85 | 1.94 | 1.90 | 0.10 |
| Chr4_55141055 | 53.88 | 53.92 | 54.45 | 51.95 | 54.07 | 52.52 | 52.67 | 52.65 | 47.16 | 53.16 | 53.69 | 53.43 | 52.92 | 54.03 | 51.80 | 53.25 | 51.65 | 53.40 | 52.81 | 1.63 |
| Chr4_55593464 | 52.16 | 48.69 | 49.10 | 50.13 | 50.45 | 48.30 | 48.32 | 51.47 | 47.46 | 49.77 | 46.89 | 48.70 | 48.33 | 50.07 | 51.94 | 51.45 | 48.28 | 50.92 | 49.58 | 1.58 |
| Chr4_55599284 | 10.02 | 9.43 | 9.39 | 9.53 | 10.34 | 9.52 | 9.42 | 9.87 | 9.96 | 11.31 | 10.19 | 9.35 | 10.48 | 9.30 | 9.21 | 10.56 | 9.59 | 9.06 | 9.81 | 0.59 |
| Chr4_55602765 | 43.95 | 43.71 | 43.52 | 44.08 | 42.23 | 43.55 | 42.06 | 43.02 | 42.38 | 43.65 | 42.92 | 44.83 | 44.39 | 44.56 | 44.84 | 43.93 | 43.94 | 43.44 | 43.61 | 0.83 |
| Chr7_55211080 | 19.04 | 18.44 | 17.86 | 17.50 | 17.79 | 18.54 | 19.19 | 17.90 | 20.08 | 18.97 | 18.59 | 17.94 | 18.24 | 19.29 | 17.89 | 18.81 | 17.73 | 18.31 | 18.45 | 0.68 |
| Chr7_55211097 | 20.52 | 19.83 | 18.52 | 18.68 | 19.61 | 20.61 | 20.91 | 19.80 | 20.55 | 20.89 | 20.88 | 19.30 | 20.27 | 20.15 | 18.84 | 20.89 | 18.58 | 19.49 | 19.91 | 0.85 |
| Chr7_55233043 | 10.72 | 11.96 | 11.07 | 11.35 | 11.09 | 11.29 | 10.83 | 10.00 | 10.13 | 10.83 | 10.62 | 11.11 | 11.18 | 11.77 | 11.37 | 11.01 | 11.67 | 11.10 | 11.06 | 0.51 |
| Chr7_55241755 | 64.74 | 67.66 | 68.30 | 71.21 | 62.95 | 66.93 | 70.27 | 67.13 | 67.52 | 66.76 | 65.66 | 69.74 | 68.04 | 71.71 | 70.16 | 67.66 | 71.11 | 69.91 | 68.19 | 2.37 |
| Chr7_55249131 | 14.85 | 15.20 | 14.03 | 14.09 | 14.92 | 15.29 | 15.23 | 15.28 | 14.74 | 15.98 | 13.97 | 15.17 | 15.30 | 14.09 | 15.24 | 13.81 | 14.53 | 13.30 | 14.72 | 0.70 |
| Chr7_55249143 | 13.88 | 14.71 | 13.46 | 14.16 | 14.27 | 14.52 | 14.51 | 14.79 | 14.73 | 15.37 | 13.83 | 15.01 | 14.93 | 14.43 | 14.25 | 13.24 | 14.90 | 13.01 | 14.33 | 0.64 |
| Chr7_140453193 | 9.78 | 10.14 | 10.37 | 10.71 | 10.17 | 10.49 | 9.88 | 10.14 | 9.75 | 10.41 | 9.76 | 10.03 | 10.23 | 10.60 | 10.99 | 10.17 | 10.75 | 10.10 | 10.25 | 0.36 |
| Chr12_25378647 | 3.54 | 3.90 | 3.97 | 4.01 | 4.03 | 4.23 | 4.07 | 4.28 | 4.03 | 4.06 | 4.06 | 3.92 | 3.56 | 4.12 | 4.01 | 3.97 | 4.01 | 3.65 | 3.97 | 0.20 |
| Chr12_25380275 | 3.68 | 3.64 | 3.12 | 3.05 | 3.53 | 3.49 | 3.49 | 3.85 | 2.89 | 3.80 | 3.21 | 3.50 | 3.87 | 3.32 | 3.98 | 3.61 | 3.25 | 3.55 | 3.49 | 0.30 |
| Chr12_25398284 | 3.89 | 3.80 | 3.25 | 3.35 | 3.48 | 3.91 | 3.25 | 3.58 | 3.84 | 3.69 | 3.72 | 3.25 | 3.70 | 3.85 | 4.28 | 3.48 | 3.57 | 3.57 | 3.64 | 0.27 |
